# Supplementary material for: Assessment of Cassava Pollen Viability and Ovule Fertilizability under Red-Light, 6-Benzyl Adenine, and Silver Thiosulphate Treatments
Source: Plants (Basel). 2024 Jul 20;13(14):1988. doi: 10.3390/plants13141988 (PMC11280604; doi:10.3390/plants13141988)
Supplement: Supplementary file 1 [file plants-13-01988-s001.zip › plants-3072075-supplementary.pdf]

plants

Supplementary Materials

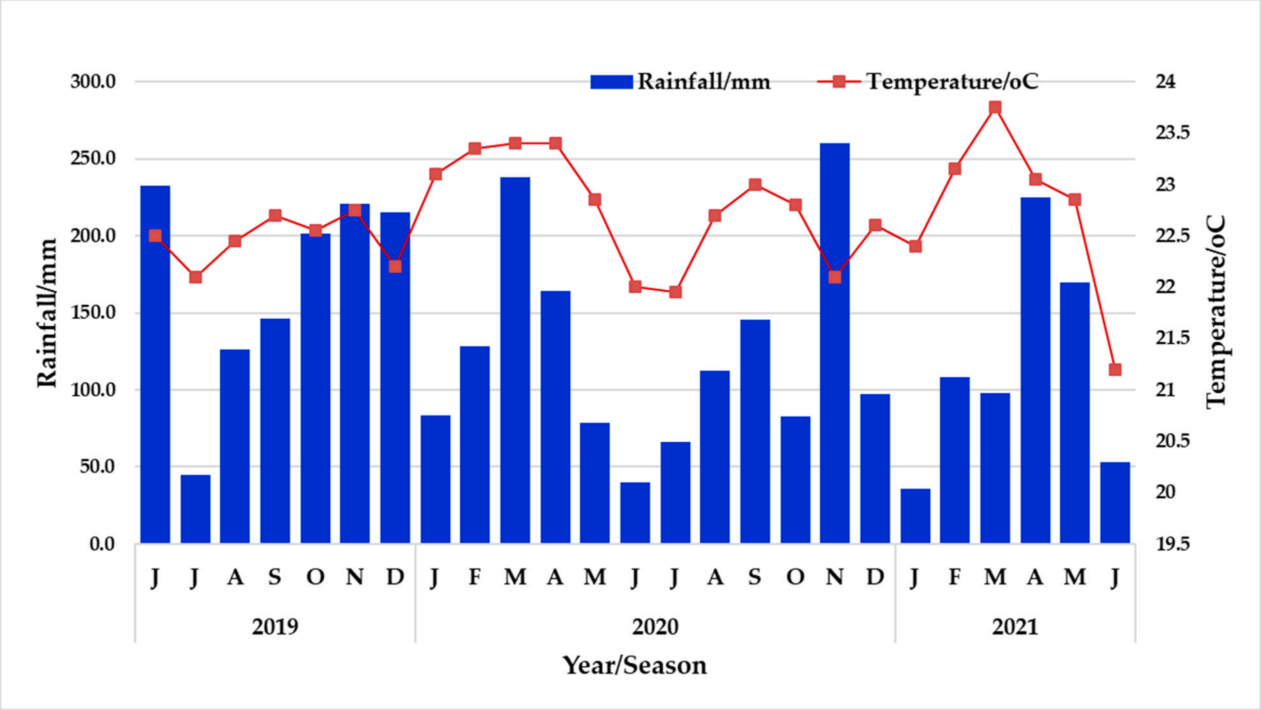

Supplementary Figure S1: Average monthly rainfall and temperature during the study period, June 2019 to June 2021. Figure adopted from [22].

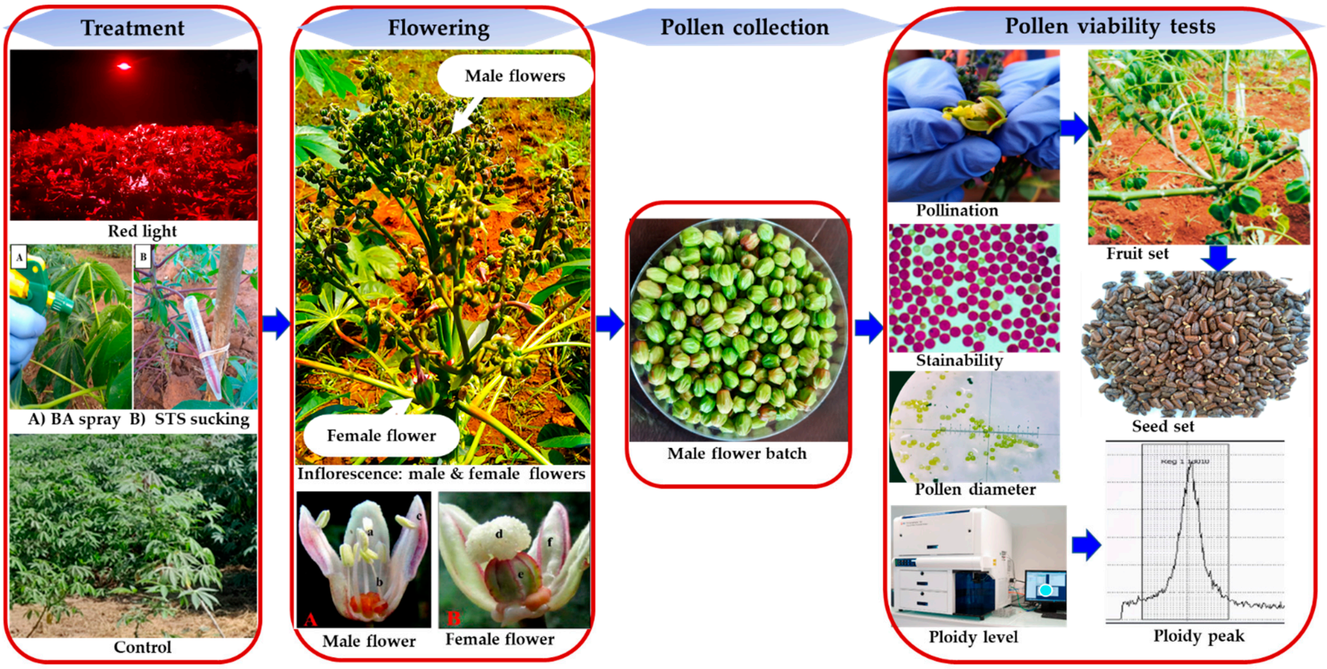

Supplementary Figure S2: Assessment of cassava pollen viability and ovule fertilizability under red-light and plant-growth-regulator treatments
